# Fermentation techniques for protein enrichment of cassava peel

**DOI:** 10.1002/jsfa.70525

**Published:** 2026-02-23

**Authors:** Aluizio Raimundo Bastos de Oliveira Júnior, Caroline Emanuelle do Amaral Santa Rosa de Oliveira, Isadora Gabriele da Silva Matos, Luis Fernando Souza Ribeiro, José Augusto de Souza Estumano, João Gabriel Sodré Lima, Cristian Faturi, Thiago Carvalho da Silva

**Affiliations:** ^1^ Institute of Health and Animal Production Federal Rural University of the Amazon (UFRA) Belém Brazil; ^2^ Faculty of Veterinary Medicine Federal University of Pará (UFPA) Belém Brazil; ^3^ School of Agricultural and Veterinary Sciences São Paulo State University (UNESP) Jaboticabal Brazil; ^4^ Institute of Agricultural Sciences Federal Rural University of the Amazon (UFRA) Belém Brazil; ^5^ Department of Animal Science and Biological Sciences Federal University of Santa Maria (UFSM) Santa Maria Brazil

**Keywords:** aeration, Manihot esculenta, mixed fermentation, non‐conventional feedstuff, *Saccharomyces cerevisiae*

## Abstract

**BACKGROUND:**

The use of agro‐industrial by‐products such as unconventional feeds in animal production is an alternative for reducing the high costs associated with conventional feeds. The present study aimed to evaluate the effect of aeration, pH adjustment, the use of yeast (*Saccharomyces cerevisiae*) and the amount of urea on the enrichment of cassava peel (CPe) by mixed fermentation and their impacts on chemical composition, protein fractionation, nutrient cost and yield. Two experiments were conducted. Experiment 1 evaluated the effects of aeration (A) and pH adjustment (pHad). Experiment 2 assessed the effects of yeast inoculation (Y) and urea dose (U). Yeast treatments consisted of either inoculation at 50 g kg^−1^ CPe or no inoculation, while urea was applied at 60 g kg^−1^ CPe (U60) or 120 g kg^−1^ CPe (U120).

**RESULTS:**

In Experiment 1, an interaction (*P* < 0.05) was observed on the concentration of ash, ether extract, protein fraction A, fresh matter yield and dry matter cost. Aeration of the solutions applied to CPe increased crude protein (CP) concentration. In Experiment 2, no interaction was observed for CP. Separately, the U120 dose increased CP content because of higher nitrogen availability, whereas yeast inoculation also significantly increased CP, suggesting the conversion of non‐protein nitrogen into microbial true protein (yeast biomass).

**CONCLUSION:**

Mixed fermentation is effective in increasing the protein concentration of CPe. Aeration has a greater impact on the protein content than pH adjustment. Furthermore, the urea dose has a greater impact on protein content than the yeast inoculation in the enrichment process of non‐aerated CPe. © 2026 The Author(s). *Journal of the Science of Food and Agriculture* published by John Wiley & Sons Ltd on behalf of Society of Chemical Industry.

## INTRODUCTION

Cassava peel (CPe) is a by‐product that can serve as a complete or partial substitute for maize in animal production systems because of its high starch content, ranging from 48.0% to 56.1%.^1^, ^2^ Its utilization aims to reduce feed costs and to mitigate environmental pollution resulting from inappropriate disposal.^3^ Approximately 220 kg and 350 kg of peels are generated through manual and mechanical processing, respectively, for every metric ton of processed cassava root.^4^ This residue is produced during the initial stages of cassava processing and comprises the peel, periderm and root scraps.^5^

The CPe typically contains 29.6% dry matter (DM), 16.8% ash, 2.1% crude protein (CP) and 52.1% neutral detergent fiber^6^ (NDF). As a result of its high energy content with a metabolizable energy of 2.86 Mcal kg^−1^ DM^7^ and low protein concentration, recent research has focused on its use as a substrate for microbial cultivation to enhance its nutritional value, particularly by increasing microbial protein content.^8^ This approach aims to partially replace conventional protein sources such as soybean meal.^9^, ^10^, ^11^

Microbial protein, commonly referred to as single cell protein, consists of dried biomass from fungi, bacteria, yeasts and algae.^11^, ^12^ These microorganisms can be cultivated on many agro‐industrial residues, including fruit pulp, vegetable peels, bran, straw and forage palm.^13^, ^14^ Yeasts are suitable for single‐cell protein production as a result of their high protein content and low levels of nucleic acids, and they are commonly used in traditional fermentation processes for human food.^11^, ^15^

Protein enrichment of cassava chips (dehydrated root) using a combination of solid‐state fermentation and liquid/submerged‐state fermentation has been shown to increase the substrate's protein content by up to ten‐fold.^8^ Polyorach *et al*.^10^ reported CP levels ranging from 33.0 to 47.5% on a DM basis following enrichment by solid‐state fermentation and liquid/submerged‐state fermentation.

In feeding trials with goats, CPe enriched through microbial fermentation successfully replaced up to 50% of the concentrate in diets formulated with 14% CP, without compromising nutrient utilization or productive performance.^6^ Similarly, Boonnop *et al*.^16^ demonstrated that the complete replacement of soybean meal with cassava root enriched with yeast and urea (30% CP) in dairy heifer diets enhanced ruminal fermentation efficiency, microbial protein synthesis and nutrient utilization.

Despite the promising potential of protein enrichment techniques, limited data are available regarding the composition of protein fractions of enriched CPe. Furthermore, the lack of standardization and reproducibility remains a significant challenge, as reported values vary widely across studies.^8^, ^10^ Therefore, optimizing ingredient ratios and fermentation protocols may be effective strategies for reducing production costs while maintaining consistent protein levels.

In this context, it was hypothesized that different CPe enrichment methods influence crude protein concentrations and differ in their associated costs. The objective of the present study was to evaluate the effects of aeration, pH adjustment, yeast inoculation (*Saccharomyces cerevisiae*) and urea supplementation on the mixed fermentation process of CPe, with the aim of increasing its protein content.

## MATERIALS AND METHODS

### Treatments and experimental design

The experiment was conducted at the Animal Science Sector of the Federal Rural University of Amazonia (UFRA), located in Belém, Pará (1°27′13.8″S 48°26′00.3″W). The area is situated within the Amazon biome, featuring an average annual temperature of approximately 28 °C. The general climatic conditions (hot and humid) classify the region as having a tropical climate, Köppen classification Af.^17^, ^18^ The CPe was obtained from a local establishment in the municipality of Santa Maria do Pará, Pará, with an average price of US$ 0.04.

Two experiments were conducted to evaluate the enrichment of CPe through mixed fermentation with yeast. Both experiments comprised four treatment combinations arranged in a 2 × 2 factorial design within a completely randomized design. In Experiment 1, the first factor was aeration (A), with two levels: with and without aeration. the second factor was pH adjustment (pHad), with treatments either adjusted to pH 4.5 or left unadjusted (natural pH of 5.3).

Experiment 2 was carried out under conditions without aeration or pH adjustment. The first factor was yeast inoculation (Y), with two levels: inoculation at 50 g kg^−1^ CPe and without inoculation. The second factor was urea dose (U), applied at either 60 g kg^−1^ CPe (U60) or 120 g kg^−1^ CPe (U120), both based on fresh matter. Each treatment was replicated six times, resulting in a total of 24 experimental units.

### Enrichment process

To conduct the experiment, the ingredients were sourced from various commercial establishments. For the protein enrichment of the CPe, the materials used were yeast, urea, refined sugar and cane molasses. The yeast (Instant Dry Yeast; Angel Yeast Co., Ltd, Cairo, Egypt), refined sugar and urea were purchased from local vendors in Belém, Pará. The enrichment process was adapted from the methodologies described by Boonnop *et al*.^8^ and Polyorach *et al*.,^10^ with the key procedures outlined below.

#### Experiment 1

To activate the yeast, 50 g of yeast was weighed and placed into a flask, followed by the addition of 50 g of cane sugar and 250 mL of distilled water. The mixture was stirred thoroughly and incubated at room temperature for 1 h (solution A). For the preparation of the liquid medium, 60 g of molasses was dissolved in 250 mL of distilled water, followed by the addition of 120 g of urea. The pH of the solution was then adjusted to 4.5 using H₂SO₄ (solution B). Solutions A and B were mixed at a 1:1 ratio in a 5‐L bucket. Aeration of this solution was performed for 120 h at room temperature using an air pump with a submerged diffuser, placed at the bottom of the bucket. After the aeration period, the resulting fermentation medium (the combined Solution A and Solution B) was mixed with CPe in fresh form at a ratio of 1 mL of medium for every 2 g of CPe. The resulting material was then subjected to a two‐step drying process, starting with shade‐drying for 72 h, followed by sun‐drying for an additional 48 h. The final dried product was stored in plastic bags.

#### Experiment 2

The procedure for the yeast‐inoculated treatment followed the methodology detailed in Experiment 1, with crucial modifications: urea was added according to the treatment levels (U60 or U120), the pH of Solution B was not adjusted and the combined solutions (1:1 ratio) were subjected to fermentation without aeration for 60 h. The treatment without yeast inoculation followed the same procedure, excluding the initial addition of 50 g of yeast to Solution A. Following the enrichment process, the resulting fermentation medium was mixed with CPe in fresh form. The resulting material was then subjected to a two‐step drying and storage protocols were identical to those described for Experiment 1.

### Chemical analysis

At the end of the drying process, all materials were stored in a freezer at −20 °C. For chemical composition analysis, the samples were thawed at room temperature, weighed, and subjected to pre‐drying in a forced‐air circulation oven at 55 °C for 72 h. Subsequently, the samples were ground using a Willey‐type knife mill equipped with a 1‐mm mesh sieve.

The enriched CPe was analyzed following the standard analytical procedures established by the AOAC^19^ official methods. The following parameters were evaluated: DM (method 934.01),^19^ ash (method 923.03),^19^ CP (method 978.04),^19^ NDF and acid detergent fiber (ADF, method 973.18).^19^ The concentration of ether extract (EE) was by method Am‐5‐04 according to the AOCS.^20^ The non‐fibrous carbohydrate (NFC) content was calculated based on NRC^21^ guidelines, using the equation: NFC = 1000 – CP − EE – ash − NDF.

Protein fractionation was performed according to the recommendations of Licitra *et al*.^22^ Fraction A was calculated as the difference between total nitrogen and trichloroacetic acid (TCA)‐insoluble nitrogen, as described by Sniffen *et al*.^23^

The B1 + B2 fraction was calculated using the formula B1 + B2 = %N‐insoluble TCA − %NDIN (neutral detergent insoluble nitrogen).^23^ The B3 fraction was calculated as the difference between %NDIN and %ADIN (acid detergent insoluble nitrogen) and the C fraction was equivalent to %ADIN.^23^

The composition of fresh CPe is presented in Table 1.

**Table 1:** Chemical composition and protein fractionation of fresh CPe

Item	Cassava peel (g kg^−1^ DM)
DM (g kg^−1^ fresh matter)	309.72
Ash	71.52
CP	46.89
EE	5.86
NDF	178.87
ADF	124.89
NFC	696.8

	Crude protein fractionation (g kg^−1^ CP)
Fraction A	546.50
Fraction B1 + B2	204.30
Fraction B3	146.60
Fraction C	102.60

A: non‐protein nitrogen (NPN); B1 + B2: soluble protein; B3: NDF‐bound protein C: ADF‐bound protein.

### Cost analysis

The cost analysis was conducted directly for both experiments, taking into account only the purchase prices of the ingredients utilized in the mixed fermentation process. This evaluation aimed to compare the costs associated with the different experimental treatments, excluding labor expenses and other operational costs. All costs were converted from Brazilian Reais (R$) to US Dollars (USD) using the official exchange rate for the date of 28 August 2025: R$ 5.4109 per USD 1.00.

### Statistical analysis

The chemical composition, protein fractionation and cost data of the enriched CPe were subjected to statistical analysis. Analysis of variance was performed using generalized linear mixed models via the PROC GLIMMIX procedure in SAS software.^24^

The statistical models for both experiments considered all factors and their respective interactions as fixed effects. Specifically, for Experiment 1, the model included the effects of A, pHad and their interaction, as shown in Eqn (1):

(1) $$Y_{\mathrm{ijk}}=\mu +A_{i}+{\text{pHad}}_{j}+{\left(A\times \text{pHad}\right)}_{\mathrm{ij}}+{ℇ}_{\mathrm{ijk}}$$

Similarly, for Experiment 2, the model included the effects of Y, U and their interaction, as shown in Eqn (2):

(2) $$Y_{\mathrm{ijk}}=\mu +Y_{i}+U_{j}+{\left(Y\times U\right)}_{\mathrm{ij}}+{ℇ}_{\mathrm{ijk}}$$

where *μ* is the overall mean and *ℇijk* is the residual error. Given the completely randomized design nature, the residual error *ℇijk* constitutes the sole random component of the model, and no structural random factors were included. Treatment means were compared using Tukey's multiple comparison test, adopting a significance level of alpha = 0.05 for the type I error. In the event of significant interaction, the decomposition and comparison of simple effects were performed using the SLICE option in the LSMEANS statement of PROC GLIMMIX.

## RESULTS

### Experiment 1

As shown in Table 2, a significant interaction between aeration (A) and pH adjustment (pHad) was observed only for the Ash (*P* = 0.04) and EE (*P* < 0.001). Under aerated conditions, CPe treated with the pH‐adjusted solution exhibited a higher ash content compared to CPe treated with the non‐adjusted solution. By contrast, pH adjustment had no effect on ash content when the solution was applied without aeration. Furthermore, aeration alone did not influence ash content, regardless of whether the solution had undergone pH adjustment.

**Table 2:** Chemical composition of CPe enriched by mixed fermentation, under different levels of aeration and pH adjustment

pH adjustment	Aeration		Means	SEM	*P*‐value		
	With	Without			A	pHad	A × pHad
	(DM g kg^−1^)			0.22	<0.001	0.28	0.90
With	920.02	905.38	912.70				
Without	916.16	902.35	909.26				
Means	918.09 a	903.87 b					

	Ash (g kg^−1^ DM)			0.10	0.37	<0.001	0.04
With	64.62 a	60.42 ab	62.52				
Without	55.30 b	57.04 b	56.17				
Means	59.96	58.73					

	CP (g kg^−1^ DM)			1.35	<0.001	0.80	0.20
With	601.70	493.32	547.51				
Without	569.18	515.06	542.12				
Means	585.44 a	504.19 b					

	NDF (g kg^−1^ DM)			0.45	<0.001	0.01	0.13
With	146.98	111.50	129.24 a				
Without	121.94	104.68	113.31 b				
Means	134.46 a	108.09 b					

	ADF (g kg^−1^ DM)			0.38	<0.001	0.02	0.20
With	108.70	79.33	94.02 a				
Without	89.22	73.54	81.38 b				
Means	98.96 a	76.44 b					

	EE (g kg^−1^ DM)			0.07	<0.001	0.003	0.003
With	14.40 a	11.42 b	12.91				
Without	14.32 a	7.27 c	10.79				
Means	14.36	9.34					

	NFC (g kg^−1^ DM)			1.69	<0.001	0.31	0.08
With	177.33	330.20	253.77				
Without	238.74	313.30	276.02				
Means	208.04 b	321.75 a					

Abbreviations: A × pHad, interaction; A, aeration; ADF, acid detergent fiber; CP, crude protein; DM, dry matter; EE, ether extract; NDF, neutral detergent fiber; pHad, pH adjustment; SEM, standard error means; NFC, non‐fibrous carbohydrates. Different lowercase letters show significant differences between treatments according to Tukey's test.

The EE content did not differ when CPe was treated with aerated solutions, irrespective of whether the solution had undergone pH adjustment. Under non‐aerated conditions, CPe treated with the pH‐adjusted solution exhibited higher EE content compared to CPe treated with the non‐adjusted solution. Moreover, regardless of pH adjustment, the application of aerated solutions resulted in higher EE content in the treated CPe.

Aeration of the solutions applied to the CPe significantly increased (*P* < 0.05) the concentrations of DM, CP, NDF and ADF, as well as resulting in a significantly lower (*P* < 0.001) content of NFC compared to CPe treated with non‐aerated solutions. Adjustment of the pH in the solutions applied to the CPe significantly increased the concentrations of NDF (*P* = 0.01) and ADF (*P* = 0.02) in the enriched CPe.

Table 3 presents the results of crude protein fractionation in the enriched CPe. An interaction (*P* = 0.008) between aeration (A) and pH adjustment (pHad) was observed exclusively for fraction A. The pH adjustment of the applied solutions did not influence fraction A, regardless of whether the solutions were aerated. However, under pH‐adjusted conditions, CPe treated with aerated solutions exhibited a higher fraction A compared to those treated with non‐aerated solutions. By contrast, when the solutions were not pH‐adjusted, fraction A did not differ between aerated and non‐aerated treatments.

**Table 3:** Protein fractionation of CPe enriched by mixed fermentation under different levels of aeration and pH adjustment

pH adjustment	Aeration		Means	SEM	*P* value		
	With	Without			A	pHad	A × pHad
	Fraction A (g kg^−1^ CP)			0.14	0.06	0.98	0.008
With	939.00 a	927.90 b	933.50				
Without	932.40 ab	934.50 ab	933.50				
Means	935.70	931.20					

	Fraction B1 + B2 (g kg^−1^ CP)			0.16	0.009	0.34	0.06
With	42.70	55.30	49.00				
Without	50.40	52.70	51.60				
Means	46.60 b	54.00 a					

	Fraction B3 (g kg^−1^ CP)			0.04	0.14	0.37	0.18
With	11.10	11.00	11.10				
Without	11.50	9.00	10.30				
Means	11.30	10.00					

	Fraction C (g kg^−1^ CP)			0.04	0.02	0.39	1.00
With	8.30	6.30	7.30				
Without	7.60	5.70	6.70				
Means	7.90 a	6.00 b					

Abbreviations: A × pHad, interaction; A, aeration; pHad, pH adjustment; A, non‐protein nitrogen (NPN); B1 + B2, soluble protein; B3, NDF‐bound protein; C, ADF‐bound protein; CP, crude protein; SEM, standard error mean. Different lowercase letters show significant differences between treatments according to Tukey's test.

The highest (*P* = 0.009) mean value for the combined protein fractions B1 + B2 was observed in CPe treated with non‐aerated solutions. By contrast, the highest (*P* = 0.04) mean for fraction C was found in CPe treated with aerated solutions. Fraction B3 was not significantly affected by either pH adjustment (*P* = 0.37) or aeration (*P* = 0.14).

With respect to yield and production costs, Table 4 reveals a significant interaction for both the fresh matter yield (FMY) (*P* = 0.03) and the cost per kilogram of DM (*P* = 0.004). Aeration of the solutions applied to the CPe did not significantly affect the FMY, irrespective of whether the solutions had undergone pH adjustment. Under pH‐adjusted conditions, CPe treated with non‐aerated solutions exhibited a higher FMY compared to those treated with aerated solutions. By contrast, when the solutions were not pH‐adjusted, FMY did not differ between aerated and non‐aerated treatments.

**Table 4:** Fresh matter yield and production costs per kilogram of dry matter and crude protein of CPe enriched by mixed fermentation based on the levels of aeration and pH adjustment

pH adjustment	Aeration		Means	SEM	*P*‐value		
	With	Without			A	pHad	A × pHad
	Fresh matter yield (%)			0.34	0.006	0.14	0.03
With	32.56 b	35.24 a	33.90				
Without	34.46 ab	34.84 a	34.65				
Means	33.51	35.04					

	DM cost (US$ kg^−1^ DM)			0.04	0.02	0.10	0.004
With	0.74 a	0.69 b	0.72				
Without	0.69 b	0.70 b	0.70				
Means	0.72	0.69					

	CP cost (US$ kg^−1^ CP)			0.19	0.04	0.95	0.55
With	1.19	1.38	1.29				
Without	1.23	1.33	1.28				
Means	1.21 b	1.36 a					

Abbreviations: A × pHad, interaction; A, aeration; CP, crude protein; DM, dry matter; pHad, pH adjustment; SEM, standard error means. Different lowercase letters show significant differences between treatments according to Tukey's test.

When the solutions were aerated, CPe treated with the pH‐adjusted solution exhibited a significantly higher (*P* = 0.02) cost per kilogram of DM compared to CPe treated with the non‐adjusted solution. Under non‐aerated conditions, DM cost did not differ, regardless of pH adjustment.

Additionally, when the pH of the solutions was adjusted, aerated treatments resulted in a higher DM cost than non‐aerated treatments. Conversely, aeration did not influence DM cost when the solutions were not pH‐adjusted. The highest (*P* = 0.04) mean cost per kilogram of CP was observed in CPe treated with non‐aerated solutions, which presented a greater average cost compared to the other treatments.

### Experiment 2

As shown in Table 5, a significant interaction (*P* = 0.005) between yeast inoculation (Y) and urea dose (U) was observed exclusively for DM content. Under yeast‐inoculated conditions, CPe treated with the solution containing a lower urea dose exhibited a higher DM content compared to CPe treated with the solution containing a higher urea dose.

**Table 5:** Chemical composition of CPe enriched by mixed fermentation, based on the use of yeast and urea doses

Urea dose	Yeast		Means	SEM	*P*‐value		
	With	Without			Y	U	Y × U
	(DM g kg^−1^)			0.27	<0.001	0.02	0.005
U120	895.44 b	880.85 c	888.15				
U60	907.62 a	879.58 c	893.6				
Means	901.53	880.22					

	Ash (g kg^−1^ DM)			0.11	<0.001	0.01	0.58
U120	53.97	47.08	50.52 b				
U60	58.20	49.90	54.05 a				
Means	56.08 a	48.49 b					

	CP (g kg^−1^ DM)			1.76	0.01	<0.001	0.38
U120	488.98	460.43	474.71 a				
U60	355.85	299.45	327.65 b				
Means	422.42 a	379.94 b					

	NDF (g kg^−1^ DM)			0.26	<0.001	0.85	0.72
U120	137.24	121.97	129.60				
U60	139.40	121.26	130.03				
Means	138.32 a	121.61 b					

	ADF (g kg^−1^ DM)			0.18	0.08	0.02	0.33
U120	89.74	84.22	86.98 b				
U60	99.80	85.50	94.18 a				
Means	94.77 a	86.39 b					

	EE (g kg^−1^ DM)			0.04	0.11	0.006	0.26
U120	8.42	6.70	7.56 b				
U60	9.58	9.26	9.42 a				
Means	9.00	7.98					

	NFC (g kg^−1^ DM)			1.71	<0.001	<0.001	0.18
U120	316.87	363.08	339.98 b				
U60	432.52	517.80	475.16 a				
Means	374.69 b	440.44 a					

Abbreviations: ADF, acid detergent fiber; CP, crude protein; DM, dry matter; EE, ether extract; NDF, neutral detergent fiber; NFC, non‐fibrous carbohydrates; SEM: standard error mean; U, urea dose; Y × U, interaction; Y, yeast inoculation. Different lowercase letters show significant differences between treatments according to Tukey's test.

The DM content of CPe treated with non‐inoculated solutions did not differ, regardless of whether the solution contained a higher or lower urea dose. However, irrespective of the urea dose applied, CPe treated with yeast‐inoculated solutions exhibited higher DM content compared to those treated with non‐inoculated solutions.

There was a significant yeast effect (*P* < 0.05) for Ash, NDF, ADF, NFC and CP. The inclusion of yeast in the solutions applied to the CPe promoted higher concentrations of Ash, NDF, ADF and CP, and a lower content of NFC compared to the enriched CPe produced without yeast.

The solution containing U120 promoted a significantly higher concentration of CP (*P* < 0.05) in the enriched CPe, as well as resulting in lower contents of Ash, ADF, EE and NFC. As shown in Table 6, none of the results related to the crude protein fractionation of the enriched CPe exhibited a significant interaction effect (*P* > 0.05).

**Table 6:** Protein fractionation of CPe enriched by mixed fermentation based on the use of yeast and urea doses

Urea dose	Yeast		Means	SEM	*P*‐value		
	With	Without			Y	U	Y × U
	Fraction A (g kg^−1^ CP)			0.43	<0.001	<0.001	0.10
U120	929.20	950.10	939.90 a				
U60	897.90	925.60	911.80 b				
Means	913.50 b	938.20 a					

	Fraction B1 + B2 (g kg^−1^ CP)			0.32	<0.001	<0.001	0.92
U120	54.20	34.30	44.20 b				
U60	72.80	52.30	62.60 a				
Means	63.50 a	43.30 b					

	Fraction B3 (g kg^−1^ CP)			0.12	0.06	0.01	0.38
U120	9.10	7.00	8.00b				
U60	16.40	10.80	13.60 a				
Means	12.70	8.90					

	Fraction C (g kg^−1^ CP)			0.05	0.13	<0.001	0.57
U120	8.30	7.00	7.60 b				
U60	11.80	11.20	11.50 a				
Means	10.00	9.10					

Abbreviations: A, non‐protein nitrogen (NPN); B1 + B2, soluble protein; B3, NDF‐bound protein; C, ADF‐bound protein; CP, crude protein; SEM, standard error mean; U, urea dose; Y × U, interaction; Y, yeast. Different lowercase letters show significant differences between treatments according to Tukey's test.

The highest concentration of fraction A (*P* < 0.001) was observed in the CPe treated with non‐yeast‐inoculated solutions, whereas the highest concentration of fraction B1 + B2 (*P* < 0.001) occurred in the CPe treated with yeast‐inoculated solutions. The urea dose significantly influenced (*P* < 0.05) fractions A, B1 + B2, B3 and C. Except for fraction A, all other fractions were higher in the CPe treated with solutions containing the lower urea dose.

Regarding FMY and production costs, Table 7 shows a significant interaction between Y × U for both yield (*P* = 0.002) and cost per kilogram of DM (*P* = 0.02). When yeast was included in the solution, the CPe treated with the higher urea dose exhibited greater FMY compared to those treated with the lower dose. By contrast, when yeast was not inoculated, FMY did not differ between the higher and lower urea doses.

**Table 7:** Fresh matter yield and production costs per kilogram of dry matter and crude protein of CPe enriched by mixed fermentation based on the use of yeast and urea doses

Urea dose	Yeast		Means	SEM	*P‐value*		
	With	Without			Y	U	Y × U
	Fresh matter yield (%)			0.29	<0.001	<0.001	0.002
U120	33.28 a	33.79 a	33.54				
U60	31.03 b	33.24 a	32.14				
Means	32.16	33.52					

	DM cost (US$ kg^−1^ DM)			0.12	<0.001	<0.001	0.02
U120	0.74 a	0.67 b	0.70				
U60	0.60 c	0.50 d	0.55				
Means	0.67	0.58					

	CP cost (US$ kg^−1^ CP)			0.18	0.33	0.003	0.32
U120	1.53	1.42	1.47 b				
U60	1.65	1.65	1.65 a				
Means	1.59	1.54					

Abbreviations: CP, crude protein; DM, dry matter; SEM, standard error mean; U, urea dose; Y × U, interaction; Y, yeast inoculation. Different lowercase letters show significant differences between treatments according to Tukey's test.

Yeast inoculation of the solution did not affect FMY when the CPe was treated with the solution containing the higher urea dose. However, when the CPe was treated with the solution containing the lower urea dose, FMY was higher in the CPe treated with the non‐inoculated solution compared to the inoculated one.

Regardless of yeast inoculation, the highest DM costs were associated with the CPe treated with the solution containing the higher urea dose compared to those treated with the lower dose. Similarly, independent of the urea dose used in solution preparation, the highest DM costs were observed in the CPe treated with yeast‐inoculated solutions compared to those treated with non‐inoculated solutions.

Regarding CP cost, a significant effect (*P* = 0.003) was observed only for the urea dose. The CPe treated with solutions containing the lower urea dose exhibited a higher average CP cost compared to those treated with the higher dose.

## DISCUSSION

The results of the present study highlight that the mixed fermentation enrichment process can increase the CP concentration of CPe. However, this process also leads to significant changes in other nutrients, thereby influencing the final composition of the material.

The increase in DM content is directly associated with the dehydration process to which the enriched CPe from both experiments were subjected. In Experiment 1, the CPe treated with the aerated solution exhibited the highest DM content. The aeration setup may have contributed to the evaporation of part of the final solution applied to the CPe. Abdelrahman & Boyd^25^ reported a 92% increase in evaporation from aerated tanks using mechanical aerators. Similarly, Helfer *et al*.^26^ observed greater water evaporation with the bubbler mechanism compared to non‐aerated conditions, with evaporation rates proportional to the airflow used.

In Experiment 2, DM content was lower when yeast was not added to the solution, probably as a result of the reduced microbial biomass. Noomhorm *et al*.^27^ stated that changes in the fungi growth, changes in carbon metabolism and changes in relative humidity would affect moisture content. This behavior aligns with findings by Oboh,^9^ who tested CPE fermentation with different microorganisms and found that yeast‐inoculated peels had a DM of 93.6%, whereas non‐inoculated peels had a DM of 94.3%.

The DM content in Experiment 2 may also be influenced by urea, which possesses hygroscopic properties. A higher urea dose may retain more moisture in the peel, slow dehydration, or absorb environmental moisture, resulting in lower DM. Werner^28^ demonstrated that urea can absorb more than 100% of its own weight in water. Polyorach *et al*.^10^ similarly observed lower DM concentrations in peels enriched with higher urea doses. Notably, none of the solutions in Experiment 2 were aerated and overall DM content was lower than in Experiment 1, further supporting the role of aeration in promoting evaporation.

Ash was found in greater quantities in the CPe treated with the aerated, pH‐adjusted solution. This environment favors yeast growth, and the increase in Ash may be attributed to the consumption of organic matter (MO), NFC and ammoniacal nitrogen, which serve as carbon and nitrogen sources for yeast metabolism. However, the Ash concentration observed in this study exceeded values reported by Polyorach *et al*.^10^

In Experiment 2, Ash was influenced independently by both yeast inoculation and urea dose. Ash was higher in CPe treated with yeast‐inoculated solutions, likely a result of MO consumption. Interestingly, Polyorach *et al*.^29^ found the opposite: ash decreased when cassava root was inoculated with microorganisms, although this behavior was not discussed. Regarding urea dose, CPe treated with higher urea concentrations had lower Ash content, possibly because of increased moisture facilitating mineral leaching, comprising a trend also noted by Polyorach *et al*.,^10^ although similarly left unexplored.

The increase in CP can be attributed to both urea addition and yeast growth, representing a combination of non‐protein nitrogen (NPN) and true microbial protein. In both experiments, urea was the primary factor driving the rise in CP content compared to the original material. In Experiment 1, the consumption of NFC by yeast promoted cell multiplication and protein contribution from microbial biomass. *Saccharomyces cerevisiae* utilizes various carbon sources such as glucose, maltose, fructose, sucrose, galactose and raffinose for growth.^30^

The observed increase in NDF and ADF does not necessarily indicate more fiber content; instead, it reflects a concentration effect. As NFC is consumed by the yeast, the fibrous components become proportionally more concentrated. In CPe treated with aerated solutions, these results are consistent with yeast metabolism: aerobic respiration yields 18 ATP per glucose molecule compared to just 2 ATP via fermentation, thereby enhancing microbial biomass development.^31^ Pilajun & Wanapat^32^ reported similar findings when enriching cassava pulp with yeast, molasses, and urea, although their CP content was only 133 g kg^−1^ DM, which is approximately four times lower than the result obtained in the present study. This discrepancy is likely a result of the differing concentrations of urea and yeast used in their methodology.

In Experiment 2, CP concentration was affected independently by yeast inoculation and urea dose. The lowest CP content was found in CPe treated with non‐inoculated solutions, likely a result of the absence of microbial biomass and consequently, the lack of protein contribution from the 50 g kg^−1^ yeast dose. This pattern mirrors the findings of Polyorach *et al*.,^29^ who enriched cassava root without aeration and recorded a CP content of 35 g kg^−1^ DM in non‐inoculated chips, which is approximately 11 times lower than the value observed in the present study.

The highest CP content occurred in peels treated with the highest urea dose, reflecting the greater protein equivalent provided by urea. Nonetheless, the CP content in peels enriched with the lower urea dose was comparable to that reported by Boonnop *et al*.,^8^ who enriched cassava chips with 120 g kg^−1^ of urea and achieved a CP content of 325 g kg^−1^ DM. This result also aligns with Polyorach *et al*.,^29^ who enriched non‐aerated cassava chips with 215 g kg^−1^ of urea and observed a CP content of 421 g kg^−1^ DM. Urea is an organic compound with a high nitrogen concentration (46%) and is recognized as the most common source of NPN in animal feed, valued for its low cost and high protein equivalent (281%).^33^

The highest EE concentration was observed in the CPe treated with the aerated and pH‐adjusted solution. Similar to the behavior observed for ash, this increase may be attributed to a proportional rise because of NFC consumption or to the increase in microbial biomass. The plasma membrane of yeasts is primarily composed of lipids and proteins.^34^ Ergosterol is the main sterol essential for yeast growth and adaptation to environmental stress.^35^ Ergosterol production is oxygen‐dependent.^36^ Polyorach *et al*.^10^ enriched cassava chips under similar conditions to those in Experiment 1 and reported an EE concentration of 73 g kg^−1^ DM, whereas Polyorach *et al*.,^29^ using non‐aerated and non‐adjusted pH conditions, observed an EE concentration of 53 g kg^−1^ DM.

In Experiment 2, the lowest EE concentration occurred in the CPe containing U120, likely the result of a dilution effect caused by the elevated CP content. This contrasts with the findings of Polyorach *et al*.,^29^ who reported an increase in EE content with rising urea doses.

In Experiment 1, the highest fraction A was found in the CPe treated with the aerated and pH‐adjusted solution. Favorable environmental conditions promoted yeast growth, which reduced NPN sources at the same time as increasing microbial protein contributing to this fraction. Fraction A represents NPN and includes residual urea, ammoniacal forms (NH₃ and NH₄^+^), free amino acids and small peptides.^22^ Muindi & Hanssen^37^ stated that the analysis of amino acid concentration provides a more direct indication of the origin of the CP present in the material.

In Experiment 2, the lowest mean for fraction A was observed in the CPe treated with yeast‐inoculated solutions. This reduction may reflect yeast consumption of nitrogen sources. However, unlike Experiment 1, the absence of optimal environmental conditions may have limited yeast development, failing to compensate for NPN consumption.

The lower concentration of fractions B1 + B2 in the CPe treated with aerated solutions may be explained by the higher fraction A in these peels, potentially causing a dilution effect. Conversely, the highest B1 + B2 concentrations were found in the CPe treated with yeast‐inoculated solutions and those treated with lower urea doses, which also had lower fraction A values, supporting the dilution hypothesis.

Regarding fractions B3 and C in Experiment 2, the lowest values were observed in CPe treated with higher urea doses. These results may be linked to the breakdown of lignin–hemicellulose bonds by ammonia (NH₃). Pamungkas *et al*.^38^ and Yuan *et al*.^39^ demonstrated that urea and fermentation pretreatments reduced NDF, ADF and lignin in citronella residue and rice straw. Ammonia can disrupt hydrogen bonds in cellulose, enhancing digestibility.^40^

Although the CPe treated with the aerated and pH‐adjusted solution had the highest DM content, it also incurred the highest cost per kilogram of DM. These peels also exhibited lower FMY, possibly as a result of reduced NFC content, which directly influences product cost. In Experiment 2, the pattern observed in Experiment 1 did not repeat. The highest cost was associated with peels having lower NFC content, although these did not present the lowest yield or DM content.

Regarding CP cost, both experiments revealed a clear inverse relationship between CP concentration and cost, with the lowest costs consistently observed in CPe exhibiting higher CP levels. This pattern indicates greater efficiency of the mixed fermentation process in treatments that promoted higher protein enrichment.

This finding becomes particularly relevant compared to the cost of crude protein derived from soybean meal, a widely used reference protein source in ruminant diets. The price of a 60‐kg bag of soybean meal ranges from US$ 22.18 to US$ 31.42,^41^ corresponding to a CP cost between US$ 0.92 and US$ 1.39 per kilogram. Within this context, protein‐enriched CPe should be regarded as a suitable protein source to supply ruminal nitrogen requirements and support microbial activity.

Cassava starch is composed, on average, of approximately 83% amylopectin and 17% amylose, exhibiting a lower amylose content than that typically reported for cereals such as corn (25–28%).^42^ Unlike corn starch, cassava starch presents high accessibility to microorganisms because of the absence of a pericarp, horny endosperm, and, most importantly, a protein matrix that would otherwise delay enzymatic hydrolysis.^43^, ^44^, ^45^ This rapid availability of fermentable energy supports the use of NPN sources, such as urea, in the enrichment process. The synchronization between the fast hydrolysis of cassava amylopectin and the simultaneous release of nitrogen from NPN optimizes microbial growth efficiency, thereby enhancing microbial protein synthesis in the rumen.

## CONCLUSIONS

The enrichment process effectively increases the crude protein concentration of CPe, mainly through the contribution of non‐protein nitrogen derived from urea and the incorporation of true protein originating from microbial growth, particularly yeast biomass. Among the evaluated factors, process aeration exerted a more pronounced effect on increasing crude protein content than pH adjustment to 4.5, demonstrating its greater relevance in promoting protein enrichment. Under non‐aerated conditions and in the absence of pH adjustment, urea dosage had a stronger influence on protein increment than yeast supplementation. Notably, yeast inclusion significantly increased the true protein fraction of CPe, reinforcing its role in improving the nutritional quality of the enriched product.

Fermentation conditions that favor yeast development resulted in the highest protein yields and, consequently, the lowest cost per kilogram of final crude protein. Additionally, the results indicate that pH adjustment may be dispensable in the enrichment process, without compromising protein accumulation. The enriched CPe constitutes a product capable of providing synchrony between rapidly fermentable energy and nitrogen availability, thereby sustaining ruminal microbial growth and activity. Nevertheless, further studies are required to assess the feasibility of large‐scale implementation and to evaluate the effects of the enriched CPe on animal intake, ruminal fermentation and productive performance.

## CONFLICTS OF INTEREST

The authors declare that they have no conflicts of interest.

## Data Availability

The data that support the findings of this study are available from the corresponding author upon reasonable request.
